# Associations influencing quality of life in caregivers of patients with amyotrophic lateral sclerosis: a stress-process model approach

**DOI:** 10.1007/s11136-026-04318-5

**Published:** 2026-06-23

**Authors:** Nafiye Turkmenel, Ersin Uskun

**Affiliations:** Department of Public Health, Faculty of Medicine, Suleyman Demirel University, Isparta, Turkey

**Keywords:** Amyotrophic lateral sclerosis, Caregiver burden, Quality of life, Perceived social support, Stress-process model, Informal caregivers, SF-12, ALSFRS-R

## Abstract

**Background:**

Caring for patients with amyotrophic lateral sclerosis (ALS) involves demands that reduce caregivers' quality of life. Although caregiver burden and perceived social support was conceptualized as an independent correlate of quality of life rather than a factor operating primarily through caregiver burden. This study examined these associations within a stress-process framework in which perceived social support was conceptualized as an independent correlate rather than a buffering factor.

**Methods:**

This cross-sectional analytical study included 118 informal caregivers of patients with ALS. Primary stressors were defined as patient functional status (ALSFRS-R), caregiving duration, and communication difficulty. Caregiver burden (Zarit Burden Interview) was considered a secondary stressor. Physical and mental quality of life were assessed using the SF-12, and perceived social support was measured with the Multidimensional Scale of Perceived Social Support. Hierarchical regression analyses were performed to examine associations specified in the conceptual model while controlling for caregiver sociodemographic and socioeconomic variables. Additional mediation analyses were conducted to examine whether caregiver burden mediated the relationship between perceived social support and quality of life.

**Results:**

Poorer patient functional status was significantly associated with higher caregiver burden, whereas communication difficulty showed a positive but non-significant association after adjustment for caregiver characteristics. Caregiver burden showed negative associations with both physical and mental quality of life. Perceived social support remained positively associated with quality of life after adjustment for caregiver burden and contributed additional explained variance in the models. Mediation analyses showed no evidence that caregiver burden mediated the association between perceived social support and either physical or mental quality of life.

**Conclusions:**

The findings are consistent with a stress-process framework in ALS caregiving, in which caregiver burden represents a central factor statistically associated with both caregiving stressors and quality of life, while perceived social support shows an independent association with quality of life. These findings suggest that both caregiver burden and perceived psychosocial resources may be relevant to caregiver well-being, although causal and intervention-related implications require further investigation.

**Supplementary Information:**

The online version contains supplementary material available at https://doi.org/10.1007/s11136-026-04318-5.

## Abstract (plain language summary)

Caring for a person with amyotrophic lateral sclerosis (ALS) is physically and emotionally demanding, and many caregivers experience reduced quality of life. Previous studies have examined caregiver burden and social support separately, but it is not well understood how these factors work together to influence caregivers’ well-being. This study examines how disease-related challenges, caregiver burden, and perceived social support are connected, and how these factors jointly affect the physical and mental quality of life of ALS caregivers. The study tests a conceptual model proposing that caregiving challenges increase caregiver burden, which in turn affects quality of life, while perceived social support contributes directly to quality of life rather than simply reducing stress. Worse patient functioning and communication difficulties were linked to higher caregiver burden. Higher burden was associated with poorer physical and mental quality of life. Perceived social support remained positively related to quality of life even after accounting for caregiver burden. These findings suggest that improving social support and reducing caregiver burden are both important for maintaining quality of life among ALS caregivers.

**Supplementary Information:**

The online version contains supplementary material available at https://doi.org/10.1007/s11136-026-04318-5.

## Introduction

Neurodegenerative diseases markedly impair functioning and quality of life and pose a growing global public health burden [10]. Amyotrophic lateral sclerosis (ALS), characterized by progressive motor neuron degeneration affecting movement, speech, swallowing, and respiration [33], is among the most devastating due to its rapid course, absence of curative treatment, and high mortality [5, 40, 42]. Its prevalence is estimated at 4–8 per 100,000 worldwide [30], with regional data from Türkiye suggesting comparable rates to Europe [46]. As the disease progresses, patients become increasingly dependent on family caregivers [3], who face sustained physical, emotional, social, and financial demands, including communication difficulties and uncertainty about disease progression [4, 11, 12, 24]. These stressors are consistently associated with greater caregiver burden and poorer quality of life [1, 20, 32, 34, 39, 41].

The caregiving experience in ALS can be conceptually understood through the stress-process model proposed by Pearlin and colleagues, which describes caregiving outcomes as the result of interrelated pathways linking objective stressors, subjective strain, and health-related consequences [37]. Within this framework, disease-related and caregiving-related demands constitute primary stressors, while caregiver burden represents a secondary stressor reflecting the individual’s subjective appraisal of caregiving strain. Caregiver burden has been identified as a central mechanism through which caregiving demands influence psychological and physical outcomes, including health-related quality of life [22]. Applying this model to ALS is particularly relevant given the progressive increase in care demands and the cumulative nature of caregiving stress across the disease trajectory.

Perceived social support represents another important component within the stress process. Previous studies have often conceptualized social support as a buffering factor that mitigates the negative effects of caregiving stress [12, 13, 19, 29, 44, 45]. However, emerging evidence suggests that in conditions characterized by high and unavoidable care demands, such as ALS, social support may function not only by reducing stress but also as an independent determinant of well-being and quality of life [16, 18, 27, 28, 31, 52, 53]. This distinction is theoretically important, as progressive functional decline may limit the extent to which caregiving burden itself can be reduced, whereas psychosocial resources may continue to exert direct effects on caregivers’ quality of life.

Although caregiver burden and perceived social support have both been associated with quality of life in ALS caregiving, these factors have rarely been examined simultaneously within a theoretically specified stress-process framework. Consequently, it remains unclear whether perceived social support primarily functions as a buffering factor or contributes independently to caregivers’ quality of life alongside caregiver burden.

Therefore, the present study aimed to examine the associations among primary stressors, caregiver burden, perceived social support, and both physical and mental components of quality of life within a theoretically derived stress-process model. Specifically, this study tested whether perceived social support demonstrates an independent association with quality of life alongside caregiver burden, rather than functioning solely as a buffering factor.

Within this study, the relationships underlying quality of life among caregivers of patients with ALS were examined within a conceptual framework derived from Pearlin’s stress-process model. Accordingly, the following hypotheses were formulated:

### H1

* Primary stressors, including patient functional impairment, caregiving intensity, and communication difficulties, are expected to show clinically meaningful positive associations with caregiver burden, with patient functional impairment expected to exert the strongest effect among the primary stressors*.

### H2

*Caregiver burden is expected to demonstrate moderate-to-strong negative associations (approximately β ≥ 0.30) with both physical and mental components of caregivers’ quality of life and to occupy a central position within the stress-process pathway*.

### H3

*Perceived social support is expected to demonstrate independent positive associations with physical and mental quality of life, with effects remaining significant after accounting for caregiver burden and primary stressors*.

These hypotheses were tested within the proposed stress-process framework using hierarchical regression analyses.

## Materials and methods

### Study design

This study was designed as a cross-sectional analytical study and reported in accordance with the Strengthening the Reporting of Observational Studies in Epidemiology (STROBE) checklist, version 4 [47], to ensure transparency and completeness of reporting (Online Appendix 1). The study was guided by an explanatory conceptual framework derived from the caregiver stress process model proposed by Pearlin et al. [37] Within this framework, disease- and caregiving-related demands were conceptualized as primary stressors influencing caregivers’ quality of life through caregiver burden, while perceived social support was examined as an independent determinant of quality of life rather than as a buffering factor.

**Fig. 1:**
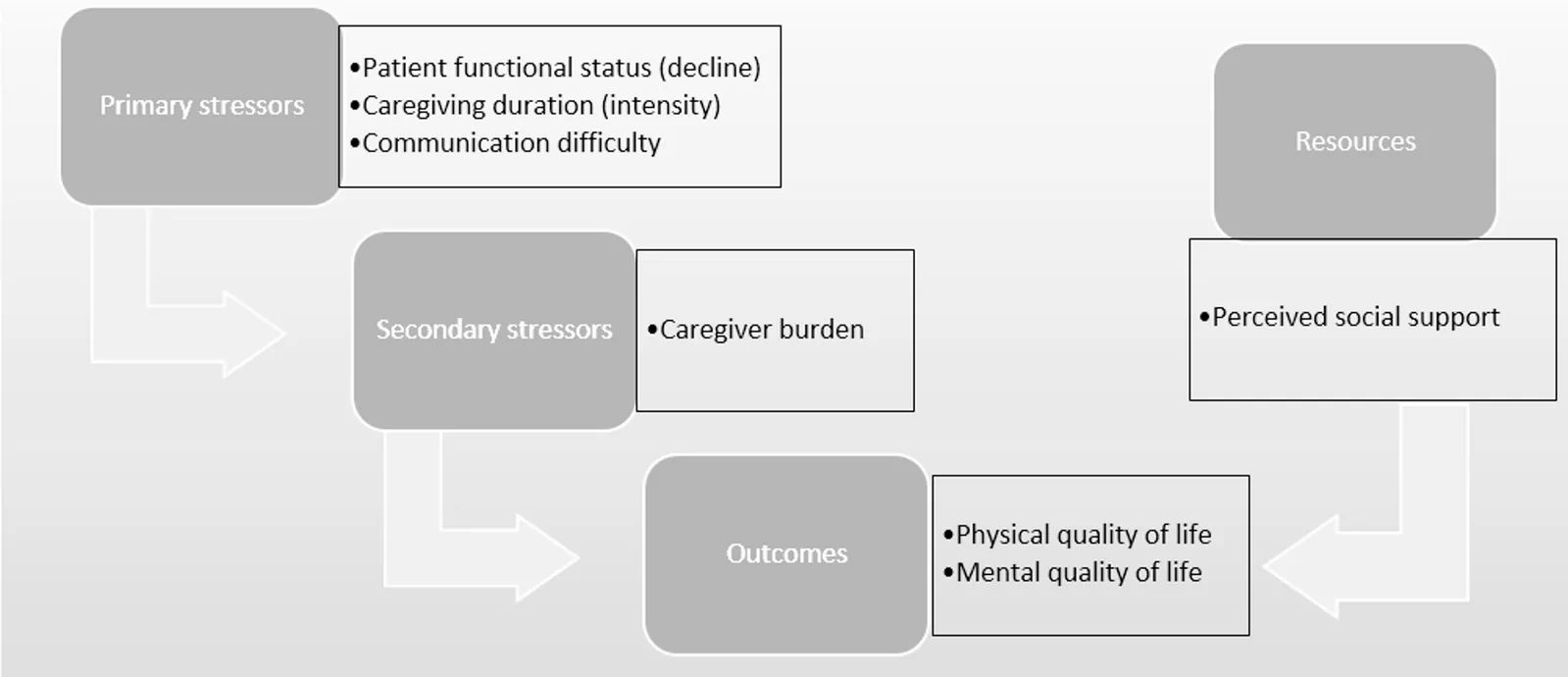
Conceptual framework of caregiver outcomes in ALS based on the stress-process model

The proposed model was based on the theoretical assumption that functional impairment, caregiving intensity, and communication difficulties are associated with caregiver burden, which in turn is statistically related to physical and mental components of quality of life. Perceived social support was examined as a protective factor contributing directly to quality of life beyond the effects of caregiver burden. Accordingly, the analytical strategy was structured to test the relationships specified in this conceptual model rather than to explore associations descriptively (Fig. 1).

Given the cross-sectional nature of the study, analyses were limited to examining associations between model components and the explanatory structure of the proposed stress-process framework, without implying causal relationships.

### Participants and data collection

Sample size was calculated using G*Power (version 3.1.9.7) based on hierarchical multiple linear regression, focusing on the detection of incremental variance (ΔR^2^) attributable to the final block of predictors. The calculation was performed using the F test for linear multiple regression (fixed model, R^2^ increase), assuming a significance level of 0.05 and a statistical power of 0.80. A small-to-moderate incremental effect size was assumed for the additional contribution of perceived social support beyond primary stressors and caregiver burden (f^2^ = 0.05). Considering up to seven predictors entered in the previous blocks and one predictor tested in the final block, the minimum required sample size was estimated as 114 participants. The final sample of 118 caregivers therefore provided adequate statistical power to detect a meaningful increase in explained variance attributable to the protective factor within the proposed stress-process model.

The study population consisted of family members providing primary care to patients with ALS who were members of the ALS Motor Neuron Disease Association and agreed to participate. An interviewer-administered approach was preferred because assessment of the ALS Functional Rating Scale–Revised (ALSFRS-R) required direct interaction to ensure accurate evaluation and recording of patients’ functional status. An announcement was disseminated through the Association, and caregivers who consented to participate were interviewed. Participants were interviewed while residing in their homes in different geographical regions of Türkiye. Depending on accessibility, interviews were conducted either face-to-face during home visits or online via video calls. All interviews were conducted in Turkish by a single interviewer (author NT), a healthcare professional experienced in administering the study instruments and evaluating the ALSFRS-R. Of the 1,300 ALS patients registered with the Association, 130 caregivers (10.0%) responded to the announcement, 123 (9.5%) consented to participate, and 118 (9.1%) met the eligibility criteria. Thus, the study achieved 92.2% of the calculated sample size.

ALS patients under care were ≥ 18 years of age, diagnosed according to the revised El Escorial criteria [6], and in regular contact with the Association. Data were collected between November 15, 2023, and May 15, 2024. Sociodemographic characteristics of caregivers (age, gender, education, income, employment status, kinship, cohabitation status, and caregiving duration) and patients (age, gender, employment status, disease onset type, disease duration, ALS Functional Rating Scale–Revised [ALSFRS-R] score [7], and disease stage) were recorded. ALS stages were determined based on clinical evaluation and patient records using the King’s Staging System [38].

Caregivers completed the Zarit Burden Interview (ZBI), the Multidimensional Scale of Perceived Social Support (MSPSS), and the Short Form-12 Health Survey (SF-12), respectively, during face-to-face or online video interviews, and responses were documented by a single researcher. The total interview duration was approximately 30–45 min per dyad.

### Inclusion and exclusion criteria

Participants were eligible for inclusion if they were family members of ALS patients registered with the ALS Motor Neuron Disease Association, served as the patient’s primary caregiver, were 18 years of age or older, and voluntarily agreed to participate in the study. Individuals were excluded if they were not the primary caregiver of an ALS patient within the Association, were not a family member of the patient, were younger than 18 years, or declined participation.

### Variables

Study variables were defined and categorized according to the proposed stress-process conceptual model. In this framework, disease- and caregiving-related demands were conceptualized as primary stressors, caregiver burden as a secondary stressor, quality of life as the outcome, and perceived social support as a protective factor. Selected caregiver characteristics were included as control variables.

Primary stressors included indicators reflecting disease severity and caregiving demands. Functional impairment of the patient was assessed using the ALS Functional Rating Scale–Revised (ALSFRS-R), with lower scores indicating greater functional limitation. Caregiving intensity was represented by caregiving duration, recorded in months. Communication difficulty was assessed as a caregiver-reported dichotomous variable (no/yes) reflecting caregivers’ subjective difficulty in communicating with the patient during daily caregiving interactions, independent of the ALSFRS-R functional assessment.

Secondary stressor was defined as caregiver burden, measured using the Caregiver Burden Scale (Zarit Burden Interview, ZBI) total score, with higher scores indicating greater perceived burden.

Outcome variables were caregivers’ physical and mental quality of life, assessed using the SF-12. The Physical Component (PC) and Mental Component (MC) scores were analyzed separately, with higher scores indicating better quality of life.

Protective factor was perceived social support, measured using the Multidimensional Scale of Perceived Social Support (MSPSS) total score, where higher scores reflect higher perceived support.

Control variables included caregiver age (years), gender (male/female), education level (≤ 8 years / ≥ 9 years), and cohabitation status with the patient (no/yes), as these variables were considered potential confounders in the relationship between caregiving processes and quality of life. Monthly income per person was additionally included as a socioeconomic control variable because of its potential association with caregiver burden and quality of life.

### Instruments

*ZBI*: Originally developed by Zarit and Zarit [51], and adapted into Turkish by İnci and Erdem [26], this 22-item scale evaluates the stress experienced by caregivers of dependent individuals. Each item is rated on a 5-point Likert scale, with total scores ranging from 0 to 88. Higher scores indicate greater perceived burden.

*MSPSS*: Developed by Zimet et al. [54] and adapted into Turkish by Eker and Arkar [14], this 12-item scale assesses perceived social support. It consists of three subscales (Family, Friends, and Significant Other). Items are scored from 1 to 7, with higher scores reflecting greater perceived support. The maximum score is 84.

*SF-12*: Developed by Ware et al. [48] and validated in Turkish by Soylu and Kutuk [43], this instrument is a shortened version of the SF-36 [49]. It provides two summary scores: PC and MC. Higher scores reflect better health-related quality of life. The SF-12 was selected for its brevity, ease of administration, and robust psychometric properties. This scale is a widely used and validated general health-related quality of life measurement tool that allows for the effective assessment of both physical and mental health components,as such, it is particularly suitable for patient groups where respondent burden is a concern.

### Statistical analysis

All statistical analyses were conducted using SPSS version 29 [25]. The normality of continuous variables was evaluated using the Kolmogorov–Smirnov test, histogram inspection, and skewness–kurtosis values. Descriptive statistics were reported as mean and standard deviation for continuous variables and frequency and percentage for categorical variables.

Bivariate associations among model variables were examined using Pearson correlation coefficients for continuous variables and Spearman’s rho for correlations involving the dichotomous variable communication difficulty. These analyses were conducted to evaluate preliminary relationships between components of the proposed stress-process model.

To examine the hypothesized associations specified in the conceptual framework, regression analyses were performed in accordance with the predefined model structure. First, hierarchical multiple linear regression analysis was conducted to examine whether primary stressors (ALSFRS-R score, caregiving duration, and communication difficulty) were associated with caregiver burden after adjustment for caregiver sociodemographic and socioeconomic characteristics. Caregiver’s age, caregiver’s gender, caregiver’s education, cohabitation status, and monthly income per person were used as control variables.

Subsequently, hierarchical multiple linear regression analyses were performed separately for SF-12 PC and SF-12 MC quality of life. Variables were entered in blocks according to the theoretical model:

Block 1 included control variables (caregiver age, gender, education level, cohabitation status and monthly income per person);

Block 2 included primary stressors (ALSFRS-R score, caregiving duration, communication difficulty);

Block 3 included the secondary stressor (caregiver burden);

Block 4 included the protective factor (perceived social support).

This hierarchical structure allowed examination of (i) the contribution of caregiver burden beyond primary stressors and (ii) whether perceived social support explained additional variance in quality of life independently of caregiver burden. Changes in explained variance (ΔR^2^) and F change statistics were used to evaluate the incremental contribution of each block.

Model assumptions were assessed through residual analysis. Multicollinearity was evaluated using variance inflation factors (VIF), with values below 5 indicating acceptable levels. Standardized regression coefficients (β) were reported to facilitate comparison of effect sizes. A *p*-value < 0.05 was considered statistically significant.

Mediation analysis using PROCESS macro (Model 4) was conducted to examine whether caregiver burden (ZBI) mediated the relationship between perceived social support (MSPSS) and quality of life outcomes (SF-12 Physical Component Summary [PCS] and Mental Component Summary [MCS]).

### Ethical considerations

This study adhered to the Declaration of Helsinki. Ethical approval was obtained from Suleyman Demirel University Clinical Research Ethics Committee (14/227, October 31, 2023). Written informed consent was obtained from participants, ensuring confidentiality and anonymity. The researcher explained the purpose of the study, assured participants that their data would remain confidential, emphasized that participation was voluntary, and informed them that they had the right to terminate the survey or withdraw from the study at any time without giving a reason. They were assured that their participation would not affect the services they received from the association. Participants did not receive any financial or material incentives for their participation in the study.

## Results

### Sample characteristics

The study included 118 ALS caregivers (mean age 47.1 ± 11.8 years; 72.9% female). Most had ≥ 9 years of education (63.6%), 52.5% were unemployed, and 74.6% cohabited with the patient. Caregiving difficulties were reported by 89.0%, communication difficulties by 60.2%, and financial burden by 40.7%. Patients had a mean age of 48.5 ± 15.1 years and a mean disease duration of 42.3 ± 34.2 months; the mean ALSFRS-R score was 28.4 ± 16.9. Mean caregiver burden was 43.0 ± 13.9, perceived social support 51.7 ± 19.6, and SF-12 PC and MC scores were 40.5 ± 8.5 and 35.5 ± 9.5, respectively (Table 1).

**Table 1:** Sample characteristics and descriptive statistics of study variables

Characteristics	Category	n (%)	M (SD)
Total		118 (100.0)	
Caregiver’s			
Gender	Female	86 (72.9)	
	Male	32 (27.1)	
Education (year)	≤ 8	43 (36.4)	
	≥ 9	75 (63.6)	
Working status	Working	56 (47.5)	
	Not working	62 (52.5)	
Kinship status	Partner/spouse	45 (38.1)	
	Other relative	73 (61.9)	
Cohabitation status	Yes	88 (74.6)	
	No	30 (25.4)	
Stated challenges by caregiver			
Difficulty in care	Yes	105 (89.0)	
	No	13 (11.0)	
Difficulty in communication	Yes	71 (60.2)	
	No	47 (39.8)	
Financial burden of care	Yes	48 (40.7)	
	No	70 (59.3)	
Patient’s			
Gender	Female	55 (46.6)	
	Male	63 (53.4)	
Education (year)	≤ 8	44 (37.3)	
	≥ 9	74 (62.7)	
Working status	Working	37 (31.4)	
	Not working	81 (68.6)	
Site of symptoms onset	Bulbar	13 (11.0)	
	Spinal	105 (89.0)	
Caregiver’s age (year)			47.1 (11.8)
Patient’s age (year)			48.5 (15.1)
Monthly income per person ($)			393.8 (329.8)
Disease duration (month)			42.3 (34.2)
Caregiving duration (month)			33.8 (29.5)
ALSFRS-R score			28.4 (16.9)
MPSS			
Total score			51.7 (19.6)
Family Subscale			18.9 (6.9)
Friends Subscale			16.6 (6.9)
Significant Other Subscale			16.2 (8.1)
Caregiver Burden			43.0 (13.9)
SF-12 components			
Physical			40.5 (8.5)
Mental			35.5 (9.5)

ALSFRS-R: Revised form of Amyotrophic Lateral Sclerosis Functional Rating Scale; M: mean; SD: standard deviation; MSPSS: Multidimensional Scale of Perceived Social Support; SF-12: Short Form 12

As shown in Table 2, poorer patient functional status and greater communication difficulty were significantly associated with higher caregiver burden (r =  − 0.50 and r = 0.46, respectively; *p* < 0.001), whereas perceived social support was not related to burden. PC was positively correlated with functional status and social support and negatively correlated with communication difficulty and caregiver burden. MC showed a similar pattern, with stronger associations observed for caregiver burden (r =  − 0.56, *p* < 0.001) and functional status (r = 0.42, *p* < 0.001).

**Table 2:** Correlations among primary stressors, caregiver burden, perceived social support, and quality of life

Variables	1	2	3	4	5	6
Primary stressors						
1. Patient functional status (ALSFRS-R score)	1					
2. Caregiving duration (month)	− 0.45***	1				
3. Communication difficulty (0 = No, 1 = Yes)	− 0.45***	0.13	1			
Secondary stressor						
4. Caregiver burden	− 0.50***	0.23*	0.46***	1		
Protective factor						
5. Perceived social support (MSPSS total)	− 0.01	− 0.11	− 0.10	− 0.12	1	
Outcome variables						
6. Physical quality of life (SF-12 PC)	0.21*	− 0.19*	− 0.26**	− 0.46***	0.32**	1
7. Mental quality of life (SF-12 MC)	0.42***	− 0.09	− 0.40***	− 0.56***	0.24*	0.48***

Pearson correlation coefficients are presented for associations between continuous variables. Spearman’s rho coefficients are reported for correlations involving the dichotomous variable communication difficulty (0 = No, 1 = Yes). *p < 0.05, **p < 0.01, ***p < 0.001

ALSFRS-R: Revised form of Amyotrophic Lateral Sclerosis Functional Rating Scale; MSPSS: Multidimensional Scale of Perceived Social Support; SF-12 MC: Short Form 12 Mental component; SF-12 PC: Short Form 12 Physical component

### Predictors of caregiver burden

Hierarchical multiple regression analysis was conducted to examine whether primary stressors were associated with caregiver burden after adjustment for caregiver sociodemographic and socioeconomic characteristics (Table 3). The overall model was significant (R^2^ = 0.36, adjusted R^2^ = 0.32, *p* < 0.001). Poorer patient functional status was independently associated with higher caregiver burden (β =  − 0.47, *p* < 0.001). Communication difficulty showed a positive but non-significant association with caregiver burden (β = 0.16, *p* = 0.076). Caregiving duration was not significantly associated with caregiver burden (*p* = 0.459). These findings indicate that patient functional impairment remained the strongest predictor of caregiver burden even after adjustment for caregiver age, gender, education, cohabitation status, and monthly income per person.

**Table 3:** Predictors of caregiver burden: testing primary stressors in the stress-process model

Variables	B	*SE*	β	p	VIF
Primary stressors					
ALSFRS-R score	− 0.38	0.09	− 0.47	< 0.001	1.95
Caregiving duration (months)	− 0.04	0.05	− 0.08	0.459	1.94
Communication difficulty (No = 0, Yes = 1)	4.63	2.59	0.16	0.076	1.44
Constant	56.98	7.81		< 0.001	
Model statistics:					
R^2^ = 0.36; Adjusted R^2^ = 0.32; F = 7.78; p < 0.001					

Caregiver’s age (year), caregiver’s gender (male:0, female:1), caregiver’s education (≤ 8 years:0, ≥ 9 years:1, cohabitation status (no:0, yes:1), and monthly income per person ($) were used as control variables. Higher caregiver burden scores indicate greater perceived burden

### Hierarchical regression analysis predicting physical and mental quality of life

Hierarchical regression analysis was performed to identify predictors of physical quality of life (Table 4). In the first block, control variables including caregiver age, gender, education, cohabitation status, and monthly income per person explained 30% of the variance in SF-12 physical component scores (R^2^ = 0.30, *p* < 0.001). Female gender (β =  − 0.24, *p* = 0.004) and cohabitation with the patient (β =  − 0.26, *p* = 0.005) were associated with lower physical quality of life, whereas monthly income was not significantly associated with the outcome.

**Table 4:** Hierarchical regression model predicting physical quality of life (SF-12 PC)

Variables	B	*SE*	β	p	VIF
Block 1					
Control variables					
Caregiver’s age (year)	− 0.01	0.07	− 0.02	0.858	1.90
Caregiver’s gender (male:0, female:1)	− 4.46	1.50	− 0.24	0.004	1.18
Caregiver’s education (≤ 8 years:0, ≥ 9 years:1)	2.21	1.66	0.13	0.187	1.71
Cohabitation status (no:0, yes:1)	− 4.98	1.73	− 0.26	0.005	1.50
Monthly income per person ($)	0.01	0.01	0.10	0.333	1.89
Block 2					
Primary stressors					
ALSFRS-R score	− 0.07	0.06	− 0.13	0.242	1.95
Caregiving duration (months)	− 0.04	0.03	− 0.13	0.199	1.96
Communication difficulty (no:0, yes:1)	− 0.54	1.54	− 0.03	0.728	1.51
Block 3					
Secondary stressor					
Caregiver burden	− 0.20	0.06	− 0.32	0.001	1.58
Block 4					
Protective factor					
Perceived social support (MSPSS total)	0.09	0.03	0.20	0.012	1.15
Model statistics	R2	Adjusted R2	ΔR2	F change	*p* (ΔF)
Block 1	0.30	0.27	0.30	9.55	< 0.001
Block 2	0.33	0.28	0.03	1.53	0.212
Block 3	0.40	0.35	0.07	13.11	< 0.001
Block 4	0.44	0.38	0.04	6.60	0.012

Hierarchical multiple linear regression analysis. Variables were entered according to the predefined stress-process conceptual model. Higher SF-12 PCS scores indicate better physical quality of life. ALSFRS-R: Revised form of Amyotrophic Lateral Sclerosis Functional Rating Scale; MSPSS: Multidimensional Scale of Perceived Social Support; SF-12 PC: Short Form 12 Physical component

The addition of primary stressors in the second block did not significantly improve model fit (ΔR^2^ = 0.03, p = 0.212). In the third block, caregiver burden emerged as a significant negative predictor of physical quality of life (β =  − 0.32, *p* = 0.001), accounting for an additional 7% of the variance (ΔR^2^ = 0.07, *p* < 0.001). In the final block, perceived social support independently and positively predicted physical quality of life (β = 0.20, *p* = 0.012), contributing an additional 4% to the explained variance (ΔR^2^ = 0.04, *p* = 0.012). The final model explained 44% of the variance in physical quality of life.

Hierarchical regression analysis predicting mental quality of life is presented in Table 5. In the first block, caregiver-related control variables explained 22% of the variance in SF-12 mental component scores (R^2^ = 0.22, *p* < 0.001). Female gender was associated with lower mental quality of life (β =  − 0.20, *p* = 0.009), whereas monthly income per person was not significantly associated with mental quality of life.

**Table 5:** Hierarchical regression model predicting mental quality of life (SF-12 MC)

Variables	B	*SE*	β	*p*	VIF
Block 1					
Control variables					
Caregiver’s age (year)	0.08	0.08	0.10	0.311	1.90
Caregiver’s gender (male:0, female:1)	− 4.33	1.62	− 0.20	0.009	1.18
Caregiver’s education (≤ 8 years:0, ≥ 9 years:1)	2.57	1.81	0.13	0.157	1.50
Cohabitation status (no:0, yes:1)	− 2.65	1.87	− 0.12	0.159	1.50
Monthly income per person ($)	0.01	0.01	0.08	0.424	1.89
Block 2					
Primary stressors					
ALSFRS-R score	0.10	0.06	0.17	0.109	1.33
Caregiving duration (months)	0.03	0.03	0.08	0.416	1.96
Communication difficulty (no:0, yes:1)	− 1.79	1.67	− 0.09	0.286	1.51
Block 3					
Secondary stressor					
Caregiver burden	− 0.24	0.06	− 0.36	< 0.001	1.58
Block 4					
Protective factor					
Perceived social support (MSPSS total)	0.09	0.04	0.19	0.014	1.15
Model statistics	R2	Adjusted R2	ΔR2	F change	*p* (ΔF)
Block 1	0.22	0.18	0.22	6.26	< 0.001
Block 2	0.35	0.31	0.14	7.57	< 0.001
Block 3	0.44	0.39	0.09	16.70	< 0.001
Block 4	0.47	0.42	0.03	6.25	0.014

Hierarchical multiple linear regression analysis. Variables were entered according to the predefined stress-process conceptual model. Higher SF-12 MC scores indicate better mental quality of life. ALSFRS-R: Amyotrophic Lateral Sclerosis Functional Rating Scale–Revised; MSPSS: Multidimensional Scale of Perceived Social Support; SF-12 MC: Short Form 12 Mental component

The inclusion of primary stressors in the second block significantly improved the explanatory power of the model (ΔR^2^ = 0.14, *p* < 0.001), although none of the individual primary stressors remained statistically significant in the adjusted model. In the third block, caregiver burden was strongly and negatively associated with mental quality of life (β =  − 0.36, *p* < 0.001), explaining an additional 9% of the variance (ΔR^2^ = 0.09, *p* < 0.001). Finally, perceived social support remained independently associated with better mental quality of life (β = 0.19, *p* = 0.014), contributing an additional 3% to the explained variance (ΔR^2^ = 0.03, *p* = 0.014). The final model explained 47% of the variance in mental quality of life.

**Fig. 2:**
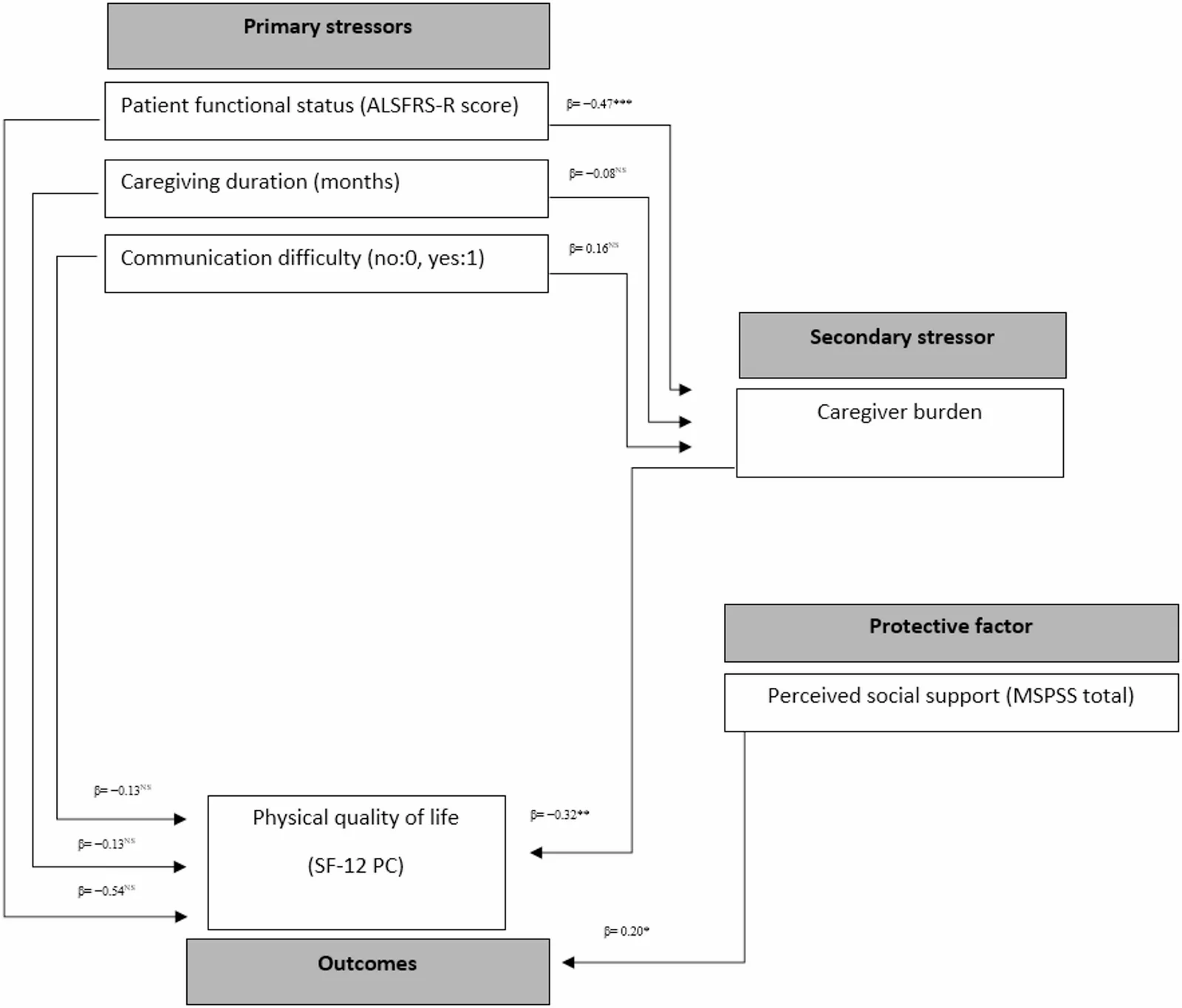
Final tested path model for physical quality of life. *Note*. Caregiver’s age (year), caregiver’s gender (male:0, female:1), caregiver’s education (≤ 8 years:0, ≥ 9 years:1, cohabitation status (no:0, yes:1), and monthly income per person ($) were used as control variables. *p < 0.05, **p < 0.01, ***p < 0.001, ^NS^ not significant. ALSFRS-R: Revised form of Amyotrophic Lateral Sclerosis Functional Rating Scale; MSPSS: Multidimensional Scale of Perceived Social Support; SF-12 PC: Short Form 12 Physical component

**Fig. 3:**
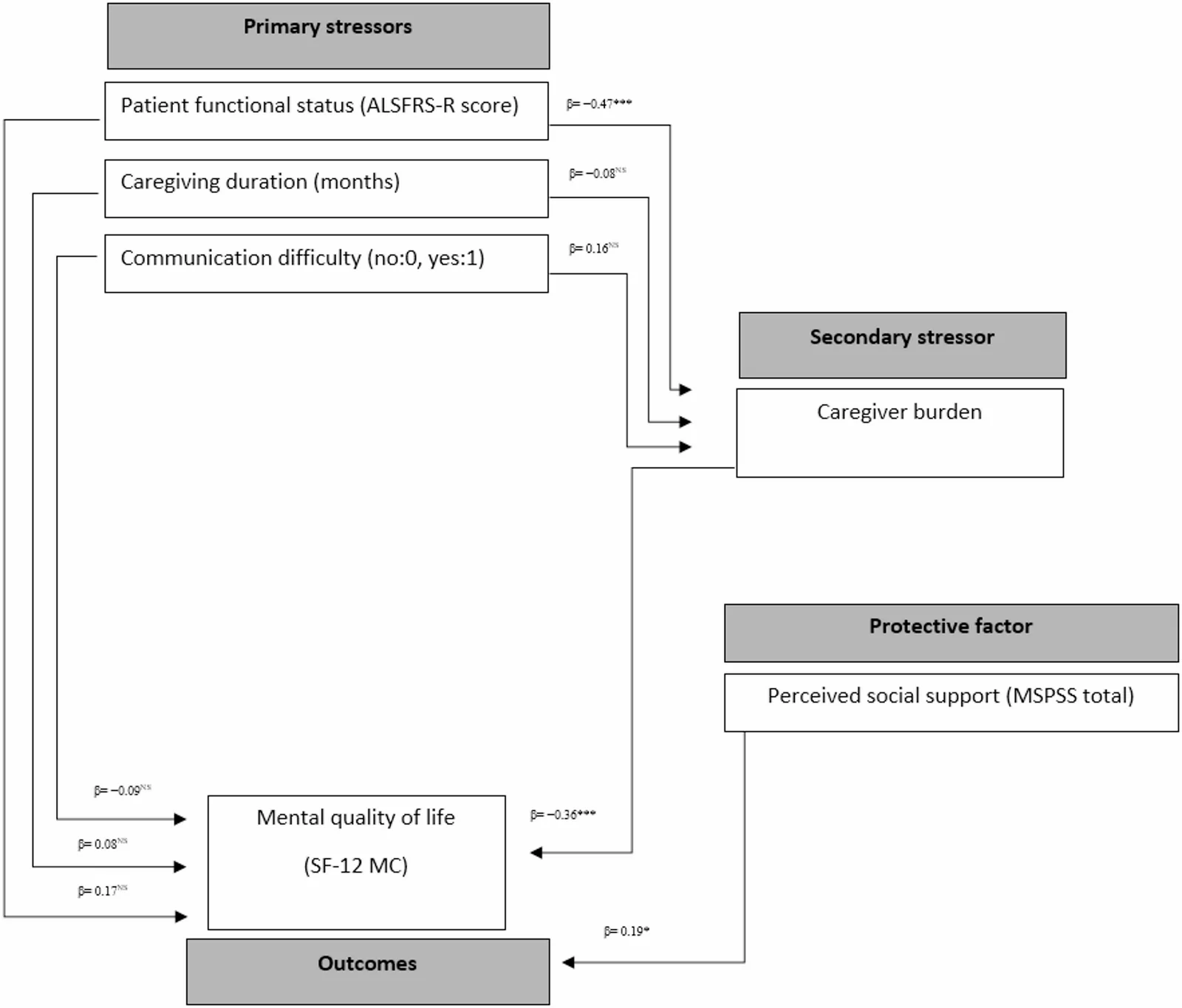
Final tested path model for mental quality of life. *Note*. Caregiver’s age (year), caregiver’s gender (male:0, female:1), caregiver’s education (≤ 8 years:0, ≥ 9 years:1, cohabitation status (no:0, yes:1), and monthly income per person ($) were used as control variables. *p < 0.05, **p < 0.01, ***p < 0.001, ^NS^ not significant. ALSFRS-R: Revised form of Amyotrophic Lateral Sclerosis Functional Rating Scale; MSPSS: Multidimensional Scale of Perceived Social Support; SF-12 MC: Short Form 12 Mental component

Figures 2 and 3 summarize the final stress-process models for physical and mental quality of life. Overall, caregiver burden emerged as the central factor associated with quality of life outcomes, whereas perceived social support demonstrated independent positive associations with both physical and mental quality of life.

In mediation analysis, for SF-12 PC, the direct effect of MSPSS on physical quality of life remained statistically significant (c′ = 0.086, 95% CI: 0.019 to 0.153), whereas the indirect effect through caregiver burden was not significant (ab = 0.017, 95% bootstrap CI: -0.051 to 0.090), indicating no evidence of mediation. Similarly, for SF-12 MC, the direct effect of perceived social support on mental quality of life was significant (c′ = 0.091, 95% CI 0.018 to 0.164), while the indirect effect via caregiver burden was not significant (ab = 0.018, 95% bootstrap CI − 0.053 to 0.098), again indicating no mediating effect of caregiver burden. Overall, results indicated a consistent pattern of a direct association between perceived social support and quality of life, without evidence of mediation by caregiver burden (Table 6).

**Table 6:** Mediation analysis of perceived social support on quality of life via caregiver burden

Outcome	Effect	B	95% CI	Interpretation
Physical quality of life (SF-12 PC)	Direct (c′)	0.086	0.019–0.153	Significant
Physical quality of life (SF-12 PC)	Indirect (ab)	0.017	− 0.051–0.090	Not significant
Mental quality of life (SF-12 MC)	Direct (c′)	0.091	0.018–0.164	Significant
Mental quality of life (SF-12 MC)	Indirect (ab)	0.018	− 0.053–0.098	Not significant

Bootstrap confidence intervals were based on 5,000 resamples. Mediation was considered significant when the 95% confidence interval did not include zero. Caregiver’s age (year), caregiver’s gender (male:0, female:1), caregiver’s education (≤ 8 years:0, ≥ 9 years:1), cohabitation status (no:0, yes:1), monthly income per person ($), ALSFRS-R score, caregiving duration (months) and communication difficulty (no:0, yes:1**)** as covariate variables

## Discussion

In this study, the relationships underlying quality of life among caregivers of individuals with ALS were examined within an explanatory framework derived from Pearlin’s stress-process model [37]. The findings suggest that health-related quality of life, as assessed by the SF-12, is not determined by a single factor but reflects a multidimensional structure in which disease-related stressors, caregiver burden, and psychosocial resources operate simultaneously. However, these interpretations are specific to the SF-12 measure, and different conclusions might have been obtained had alternative instruments assessing quality of life been used. The progressive nature of ALS renders the caregiving process complex not only in terms of physical care demands but also through psychosocial dimensions such as communication loss, role transitions, and prolonged caregiving responsibility. Accordingly, quality of life should be conceptualized as an outcome encompassing the overall caregiving experience rather than being limited to the patient’s clinical condition alone. Consistent with this perspective, previous studies have shown that quality of life among ALS caregivers is shaped by the combined influence of disease severity, caregiving demands, and psychosocial factors [2, 18, 45].

Hierarchical regression analyses revealed both shared and distinct predictors of SF-12 physical and mental health. Being female, higher caregiver burden, and lower perceived social support were associated with worse outcomes in both PCS and MCS. However, living in the same household was a significant predictor only for PCS and not for MCS. This divergence indicates that cohabitation mainly influences physical health-related quality of life, whereas mental health is shaped more by psychosocial factors.

The findings further demonstrate that caregiver burden occupies a central position within the stress-process framework. After adjustment for caregiver sociodemographic and socioeconomic characteristics, poorer patient functional status remained significantly associated with higher caregiver burden, which in turn was independently associated with lower physical and mental quality of life. Although communication difficulty showed a positive association with caregiver burden, this relationship did not remain statistically significant in the adjusted model. These findings are consistent with previous studies identifying functional decline as one of the strongest determinants of caregiver burden in ALS and suggest that this relationship persists independently of socioeconomic characteristics [29, 41, 44]. As caregiving demands intensify over the course of the disease, caregivers are increasingly exposed to physical exhaustion, psychological strain, and social restriction, all of which contribute directly to diminished well-being. Caregiver burden can therefore be understood as a central factor statistically linking primary stressors to quality of life outcomes.

A key contribution of this study lies in clarifying the role of perceived social support. Perceived social support showed an independent association with quality of life beyond caregiver burden in the present sample. Although the association between social support and quality of life is well established, the present findings confirm this relationship specifically within the context of ALS caregiving. The persistence of significant associations between perceived social support and both physical and mental quality of life after adjustment for caregiver burden as well as caregiver sociodemographic and socioeconomic characteristics suggests that perceived social support represents a distinct psychosocial correlate of caregiver well-being. While this finding is consistent with previous studies reporting positive associations between social support and quality of life [2, 23, 36, 50], it also suggests that conceptualizing social support solely as a stress-reducing mechanism may be insufficient in the context of ALS caregiving. The MSPSS measures perceived social support rather than objectively received or enacted support. Therefore, the findings of this study should be interpreted in terms of caregivers’ subjective appraisal of available support, which may differ from the actual support provided. Perceived support is shaped by individual expectations and relational experiences, and may not fully reflect assistance received from family members, friends, or formal organizations or the objective availability of support resources. In the context of ALS caregiving, support networks such as family systems and patient associations (e.g., ALS/Motor Neuron Disease organizations) may play distinct and complementary roles in shaping caregivers’ perceptions of support and overall well-being.

The lack of a significant association between perceived social support and caregiver burden may be explained by several contextual factors. In addition, the absence of a significant relationship persisted even after adjustment for monthly income, suggesting that the independent role of perceived social support cannot be explained solely by socioeconomic conditions. In societies such as Türkiye, where family-based caregiving is often regarded as a normative and moral responsibility, the caregiving role may be strongly internalized [15]. Under such conditions, receiving support may not necessarily alter perceptions of burden, as support is interpreted as part of expected family functioning rather than as a reduction in caregiving demands [8, 9]. Furthermore, perceived social support typically reflects emotional or relational resources, whereas caregiver burden is more closely linked to physical care demands and time-related constraints. As a result, informal support may enhance quality of life without substantially reducing the structural workload of caregiving. Previous literature similarly emphasizes the distinction between informal emotional support and structural or professional support in shaping caregiving outcomes [12, 21]. It should also be considered that some dimensions of perceived social support assessed by the MSPSS, particularly support from family and significant others, may partially reflect the quality and closeness of the caregiver–patient relationship itself. In ALS caregiving, interpersonal and dyadic relationship factors may therefore contribute to perceived support as well as caregiver quality of life. Previous studies have shown that psychosocial interventions, caregiver support groups, psychoeducation programs, and family-centered supportive approaches may improve perceived social support and caregiver well-being in chronic neurological conditions [13, 35]. Interventions may indirectly influence perceived social support by enhancing interpersonal and relational resources.

From a clinical perspective, these findings have important implications. Interventions in ALS caregiving have largely focused on reducing caregiver burden; however, the present model suggests that burden reduction alone may be insufficient to improve overall quality of life. Programs aimed at facilitating social participation, interpersonal connectedness, and psychosocial well-being may contribute positively to caregivers’ perceptions of support and quality of life. These benefits appear to extend beyond the effects of socioeconomic resources alone. Accordingly, comprehensive caregiver support approaches that address both caregiving burden and broader psychosocial resources may be valuable in ALS care. This perspective further supports the importance of multidisciplinary and holistic models of care in the management of ALS.

## Limitations

Several limitations should be considered when interpreting these findings. Due to the cross-sectional design, the relationships identified in the model should be interpreted as associations rather than causal pathways. All measures were based on self-report instruments, which may introduce reporting bias. As participants were recruited through a patient association, caregivers with higher levels of engagement or access to support resources may have been overrepresented, potentially limiting the generalizability of the findings. Although participants were recruited through an ALS patient organization registry and caregiver status was verified via structured interviews with both patients and caregivers, the possibility of residual selection bias cannot be entirely excluded. However, these procedures, including functional assessment using ALSFRS, substantially minimized the risk of ineligible or fraudulent participation.

In addition, the study was conducted within a single cultural context, and caregiving experiences may vary across healthcare systems and cultural environments. Future longitudinal studies are needed to examine how changes in caregiving demands, burden, and social support relate to changes in caregiver quality of life over time and to further evaluate the stability of the proposed stress-process framework.

Furthermore, the study sample may be subject to selection bias. Only a relatively small proportion of caregivers registered with the ALS Association responded to the study announcement, and participation was voluntary. Caregivers who chose to participate may have been more socially engaged, more motivated, or had greater access to psychosocial or socioeconomic resources than non-participants. This possibility is particularly relevant given the focus on perceived social support and quality of life. Nevertheless, adjustment for monthly income strengthened the robustness of the findings by reducing the likelihood that the observed associations were attributable solely to socioeconomic differences among caregivers. Furthermore, female caregivers constituted the majority of the sample, which may reflect gendered caregiving patterns but may also limit the representativeness of the findings for male caregivers. Consequently, the generalizability of the results to the broader ALS caregiver population should be interpreted with caution.

Another limitation is that cognitive and behavioral symptoms of patients with ALS were not assessed. These symptoms are known to substantially influence caregiver burden and quality of life and may have contributed to the observed associations.

## Conclusion

 Quality of life among ALS caregivers is associated with caregiver burden and perceived social support. Caregiver burden showed a consistent relationship with lower quality of life, while perceived social support was independently associated with better quality of life. As social support was assessed as perceived rather than received support, findings should be interpreted within this subjective framework. Nevertheless, the present findings highlight the potential relevance of perceived psychosocial resources within the specific context of long-term ALS caregiving, where progressive functional decline and sustained caregiving demands create unique psychosocial challenges.

## Supplementary Information

Below is the link to the electronic supplementary material.

Supplementary Material 1

## Data Availability

Data supporting this study are not openly available due to reasons of sensitivity and are available from the corresponding author upon reasonable request.
