# Supplementary material for: Associations influencing quality of life in caregivers of patients with amyotrophic lateral sclerosis: a stress-process model approach
Source: Qual Life Res. 2026 Jun 23;35(8):216. doi: 10.1007/s11136-026-04318-5 (PMC13290779; doi:10.1007/s11136-026-04318-5)
Supplement: Supplementary file 1 — Supplementary Material 1 [file 11136_2026_4318_MOESM1_ESM.docx]

**Appendix 1. STROBE Checklist for Observational Studies**

| **Section / Item** | **Item No** | **Recommendation** | **Reported on Page No** |
| --- | --- | --- | --- |
| **Title and Abstract** | 1 | Indicate the study’s design with a commonly used term in the title or the abstract | 1 |
|  | 1 | Provide in the abstract an informative and balanced summary of what was done and what was found | 1, 2 |
| **Introduction** | 2 | Explain the scientific background and rationale for the investigation being reported | 3 |
|  | 3 | State specific objectives, including any prespecified hypotheses | 4 |
| **Methods** | 4 | Present key elements of study design early in the paper | 4, 5 |
|  | 5 | Describe the setting, locations, and relevant dates, including periods of recruitment and data collection | 5 |
|  | 6 | Give eligibility criteria and sources and methods of participant selection | 5, 6 |
|  | 7 | Clearly define all outcomes, exposures, predictors, potential confounders, and effect modifiers | 6 |
|  | 8 | For each variable of interest, give sources of data and details of methods of assessment | 6 |
|  | 9 | Describe any efforts to address potential sources of bias | 6, 7 |
|  | 10 | Explain how the study size was arrived at | 5 |
|  | 11 | Explain how quantitative variables were handled in the analyses | 6, 7 |
|  | 12 | Describe all statistical methods, including those used to control for confounding | 6, 7 |
| **Results** | 13 | Report numbers of individuals at each stage of study | 7, 8 |
|  | 14 | Give characteristics of study participants and information on exposures and potential confounders | 7, 8 |
|  | 15 | Report numbers of outcome events or summary measures | 8, 9 |
|  | 16 | Give unadjusted and adjusted estimates and their precision | 8, 9 |
|  | 17 | Report other analyses performed, including subgroup or sensitivity analyses | 8, 9 |
| **Discussion** | 18 | Summarize key results with reference to study objectives | 9 |
|  | 19 | Discuss limitations of the study, taking into account sources of potential bias or imprecision | 9, 10 |
|  | 20 | Provide a cautious overall interpretation considering objectives, limitations, and relevant evidence | 10 |
|  | 21 | Discuss the generalizability (external validity) of the study results | 10 |
| **Other Information** | 22 | Give the source of funding and the role of funders | 11 |

**Note:** This checklist has been completed in accordance with the STROBE statement for cross-sectional studies.
